# Supplementary material for: Schwann cell TRPA1 mediates neuroinflammation that sustains macrophage-dependent neuropathic pain in mice
Source: Nat Commun. 2017 Dec 1;8:1887. doi: 10.1038/s41467-017-01739-2 (PMC5709495; doi:10.1038/s41467-017-01739-2)
Supplement: Supplementary file 1 — Supplementary Information [file 41467_2017_1739_MOESM1_ESM.pdf]

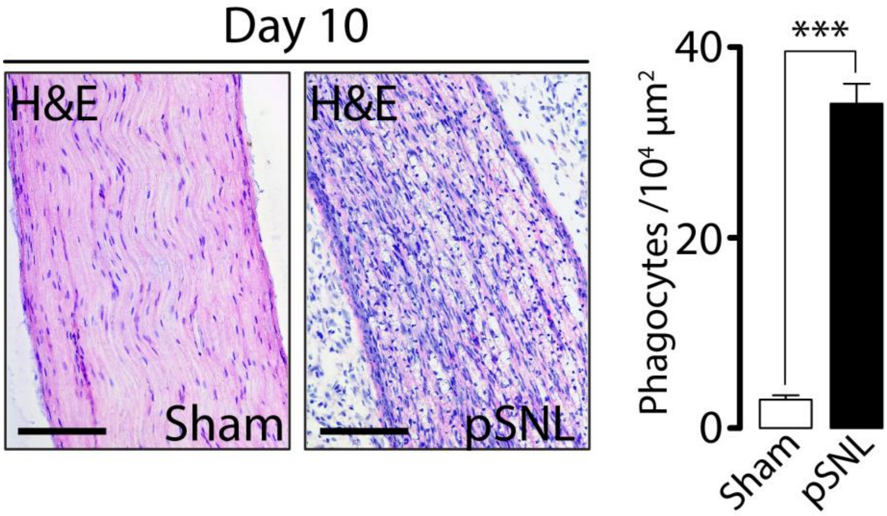

Supplementary Figure 1. Phagocytes within the injured nerve at day 10 after pSNL. Representative images of hematoxylin and eosin and number of phagocytes in the sciatic nerve trunk induced by pSNL in C57BL/6 compared to sham mice. (n=4, ***P <0.001 pSNL *vs.* Sham; unpaired two-tailed Student's t-test). Data are represented as mean±s.e.m.

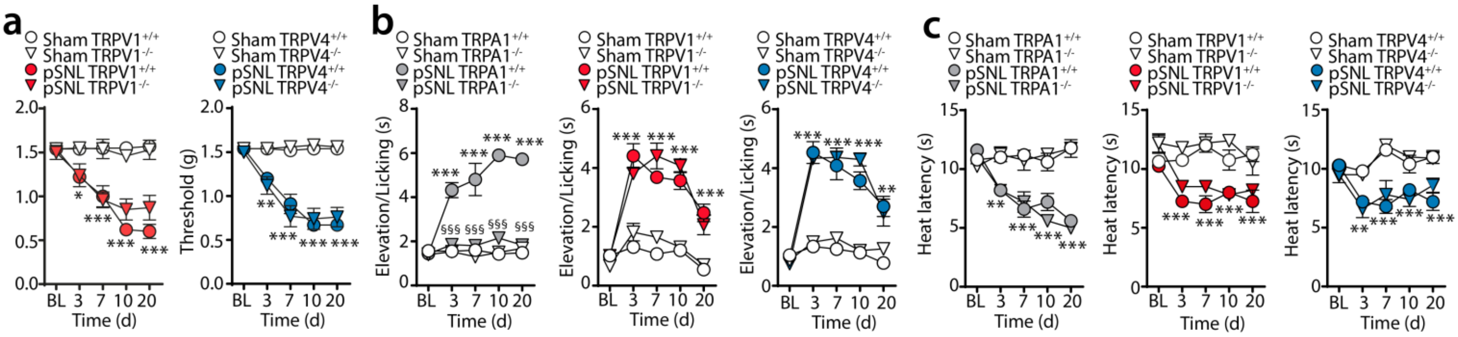

Supplementary Figure 2. TRPA1, but not TRPV1 and TRPV4, mediates pSNL-evoked mechanical and cold allodynia, while heat hyperalgesia is not mediated by that channel. (a) Mechanical, **(b)** cold and **(c)** heat hypersensitivity in sham/pSNL *Trpv1*$^{+/+}$/*Trpv1*$^{-/-}$, *Trpv4*$^{+/+}$/*Trpv4*$^{-/-}$ and *Trpa1*$^{+/+}$/*Trpa1*$^{-/-}$ mice (n=6, ***P <0.001 pSNL$^{+/+}$ *vs.* Sham$^{+/+}$; §§§P<0.001 pSNL$^{+/+}$ *vs.* pSNL$^{-/-}$; two-way ANOVA followed by Bonferroni post hoc analyses). Data are represented as mean±s.e.m.

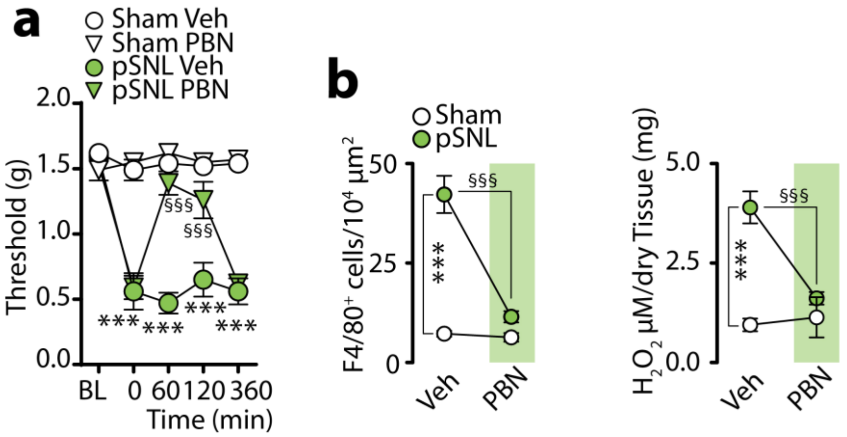

Supplementary Figure 3. Oxidative stress mediates pSNL-evoked allodynia and neuroinflammation. (a) Mechanical allodynia, and **(b)** number of F4/80$^+$ cells and H_2O_2 content in the sciatic nerve of sham/pSNL C57BL/6 mice after phenyl-N-tert-butylnitrone (PBN, 100 mg/kg, i.p.) or vehicle (veh, isotonic saline) (n=6, ***P<0.001 pSNL veh *vs.* Sham veh; §§§P<0.001 pSNL PBN *vs.* pSNL veh; two-way ANOVA followed by Bonferroni post hoc analyses and one-way ANOVA followed by Bonferroni post hoc analyses). Data are represented as mean±s.e.m.

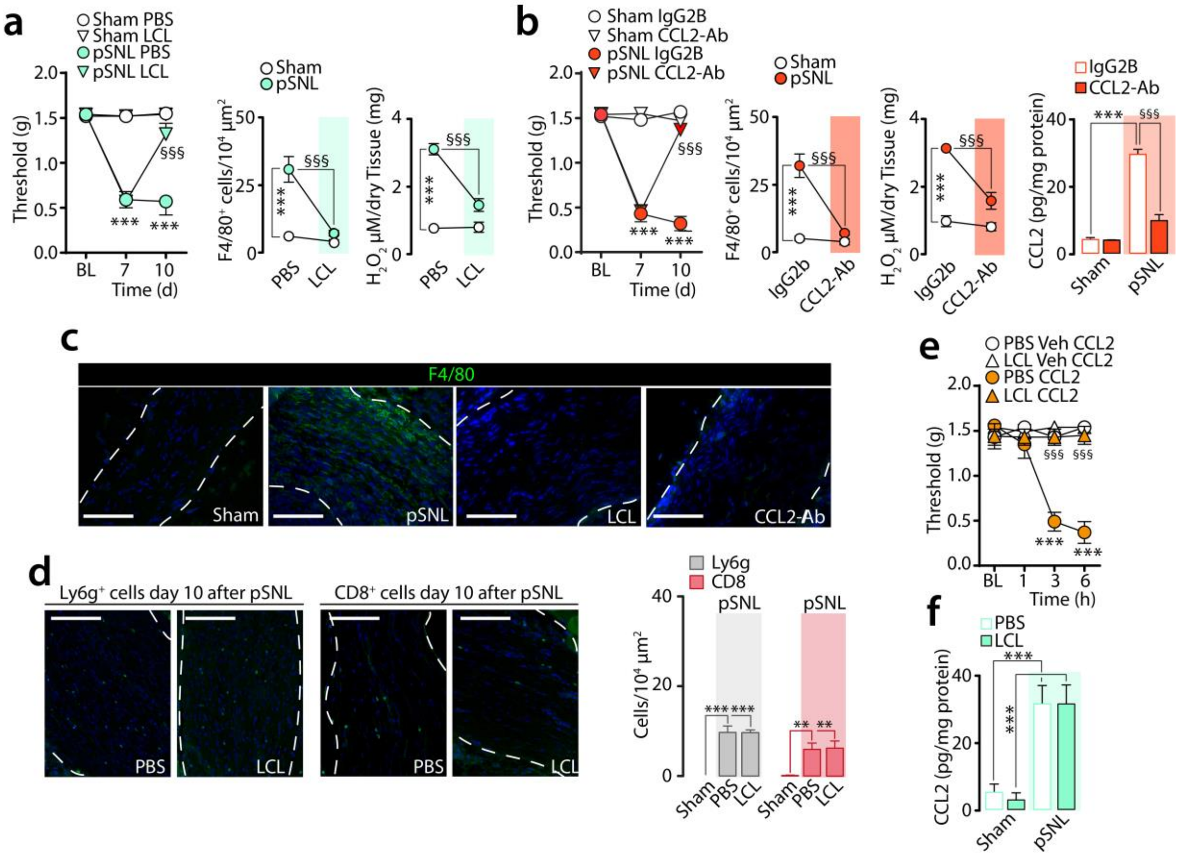

Supplementary Figure 4. Macrophage recruitment drives pSNL-evoked allodynia and neuroinflammation. (a) Mechanical allodynia, number of F4/80$^+$ cells and H_2O_2 content in the sciatic nerve of sham/pSNL C57BL/6 mice after liposome-encapsulated clodronate (LCL) or its vehicle (PBS) one injection/day (5 mg/ml, i.p.) starting from day 7 after surgery until day 10 (n=5, ***P<0.001 pSNL PBS *vs.* Sham PBS; §§§P<0.001 pSNL LCL *vs.* pSNL PBS; two-way and one-way ANOVA). **(b)** Mechanical allodynia, number of F4/80$^+$ cells, H_2O_2 content and CCL2 levels in the sciatic nerve of sham/pSNL C57BL/6 mice after one injection/day (40 µg/200 µl, i.p., from day 7 until day 10 after surgery) of CCL2-Ab or its vehicle (IgG2B) (n=5, ***P<0.001 pSNL IgG2B *vs.* Sham IgG2B; §§§P<0.001 pSNL CCL2-Ab *vs.* pSNL IgG2B; one-way and two-way ANOVA). **(c)** Representative images of F4/80$^+$ cells in the sciatic nerve of sham/pSNL C57BL/6 mice after LCL and CCL2-Ab (Scale bars: 50 µm, dashed lines indicate *perineurium*). **(d)** Representative images and number of Ly6g$^+$ and CD8$^+$ cells in the sciatic nerve of

sham/pSNL C57BL/6 mice after LCL (Scale bars: 50 µm, dashed lines indicate *perineurium*). **(e)** Mechanical allodynia induced by perineural CCL2 (1 µg) in C57BL/6 mice after LCL or its vehicle (PBS) (n=5, ***P<0.001 PBS CCL2 *vs*. Sham PBS Veh CCL2; §§§P<0.001 LCL CCL2 *vs.* PBS CCL2; followed by Bonferroni post hoc analyses). Data are represented as mean±s.e.m.). **(f)** CCL2 levels in sciatic nerves of sham/pSNL C57BL/6 mice at day 10 after LCL or its vehicle (PBS) (n=4, ***P<0.001 pSNL-PBS *vs*. sham-PBS and pSNL-LCL *vs*. sham-LCL; one-way ANOVA followed by Bonferroni post hoc analyses). Data are represented as mean±s.e.m.

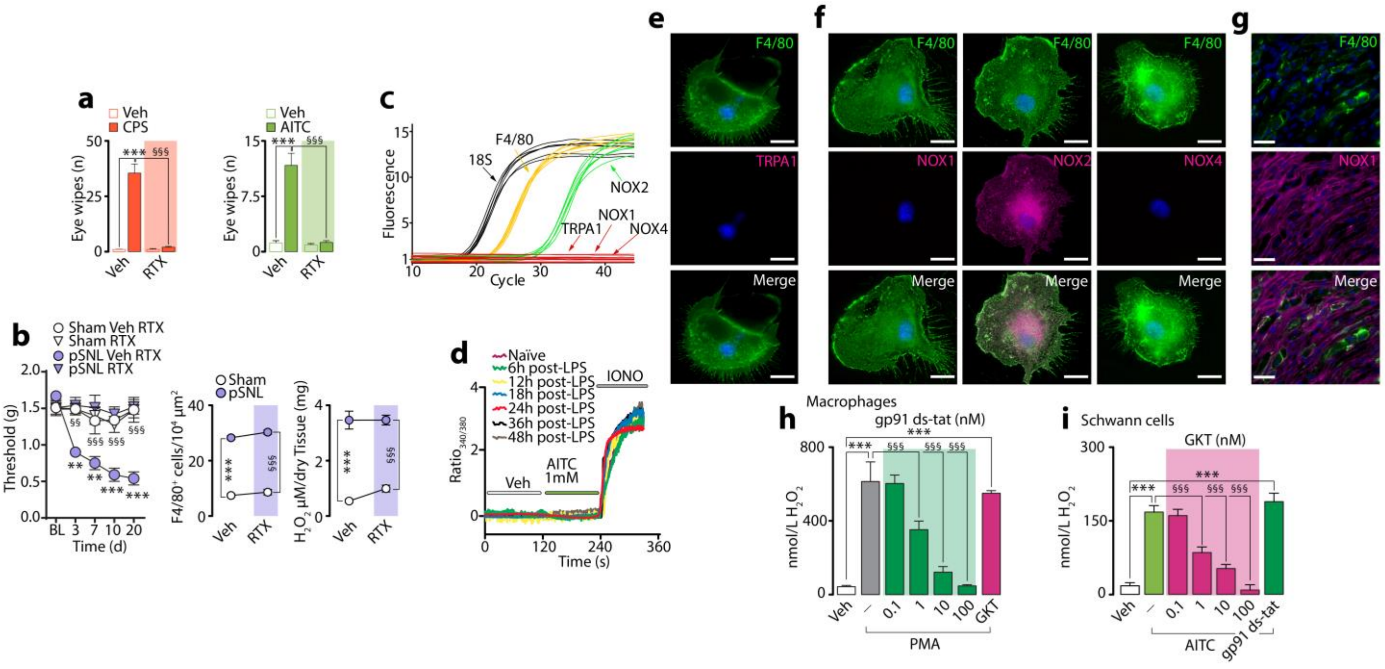

Supplementary Figure 5. Nerve fibers and macrophages do not generate the TRPA1-dependent oxidative stress. (a,b) Number of eye wipes to capsaicin (CPS, 1 nmol/5 µl) or AITC (10 nmol/5 µl), mechanical allodynia and number of F4/80^{+} cells and H_2O_2 content in the sciatic nerve of sham/pSNL C57BL/6 mice after treatment with resiniferatoxin (RTX, 50 µg/kg, subcutaneous) or its vehicle (veh, isotonic saline) (n=6, ***P<0.001 CPS, AITC *vs.* veh **(a)**, **P<0.01 and ***P<0.001 pSNL veh RTX *vs.* Sham veh RTX **(b)**; §§§P<0.001 CPS RTX *vs.* CPS veh RTX **(a),** §§P<0.01 and §§§P<0.001 pSNL RTX *vs.* pSNL veh RTX **(b)**; one-way ANOVA followed by Bonferroni post hoc analyses and two-way ANOVA followed by Bonferroni post hoc analyses). **(c)** Representative real-time PCR plot for 18S, F4/80, TRPA1, NOX1, NOX2 and NOX4 in mouse peritoneal macrophages in culture (n=3 replicates from 2 independent experiments). **(d)**, Typical traces of calcium response to AITC (1 mM) in naïve or lipopolysaccharide (LPS)-activated peritoneal macrophages. **(e)** Representative images of F4/80^{+} and TRPA1 staining in peritoneal macrophages in culture (Scale bars: 100 µm). **(f)** Representative images of NOX1, NOX2 and NOX4 and F4/80 staining in

peritoneal macrophages in culture (Scale bars: 10 μm). **(g)** Representative images of NOX1 and F4/80 staining in sciatic nerve of pSNL C57BL/6 mice. (Scale bars: 10 μm). **(h)** H_2O_2 release from cultured peritoneal macrophages by phorbol myristate acetate (PMA, 20 nM) in the presence of different concentrations of gp91ds-tat (NOX2 selective inhibitor, 0.1-100 nM) or GKT (mixed NOX1/NOX4 inhibitor, 100 nM) (n=8 replicates from 3 independent experiments, ***P<0.001 PMA *vs.* veh; §§§P<0.001 PMA gp91ds-tat *vs.* PMA veh; one-way ANOVA followed by Bonferroni post hoc analyses). **(i)** H_2O_2 release from cultured mouse Schwann cells by AITC (100 μM) in the presence GKT (0.1-100 nM) or gp91ds-tat (100 nM) (n=8 replicates from 3 independent experiments, ***P<0.001 AITC *vs.* veh; §§§P<0.001 AITC GKT *vs.* AITC veh; one-way ANOVA followed by Bonferroni post hoc analyses). Data are represented as mean±s.e.m.

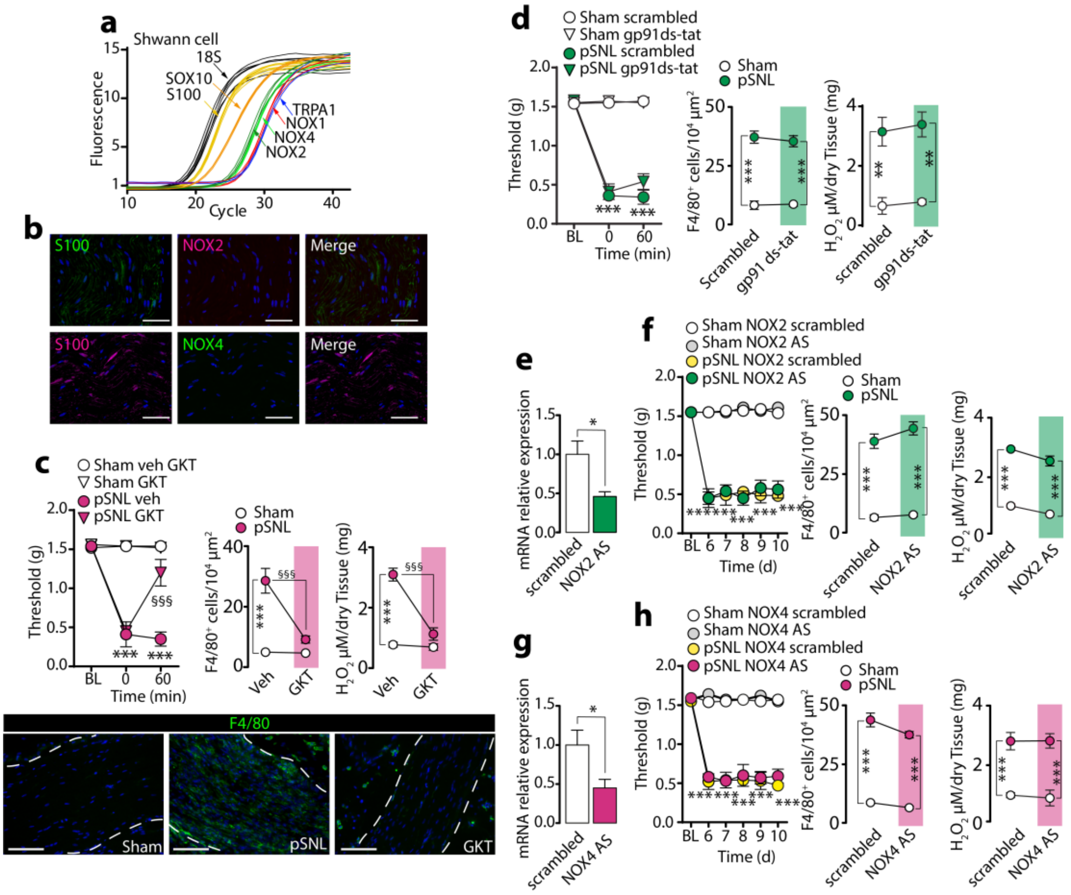

Supplementary Figure 6. NOX1, but not NOX2 or NOX4, inhibition attenuates pSNL-evoked allodynia and neuroinflammation. (a) Representative real-time PCR plot for 18S, S100, TRPA1, NOX1, NOX2 and NOX4 in cultured Schwann cells (n=3 replicates from 2 independent experiments). **(b)** Representative images of S100, NOX2 and NOX4 staining in sciatic nerve of C57BL/6 mice. (Scale bars: 50 µm). **(c)** Mechanical allodynia, representative images and number of F4/80^{+} cells, and H_2O_2 content in sham/pSNL C57BL/6 mice after the NOX1/NOX4 inhibitor, GKT137831 (GKT, 60 mg/kg, i.p.) or vehicle (veh, 4% DMSO 4% tween 80 in isotonic saline) (n=6, ***P<0.001 pSNL veh, *vs.* sham veh; §§§P<0.001 pSNL GKT *vs.* pSNL veh; two-way ANOVA followed by Bonferroni post hoc analyses and one-way ANOVA followed by Bonferroni post hoc analyses. (Scale bars: 50 µm). **(d)** Mechanical allodynia, number of F4/80^{+} cells, and H_2O_2 content in sham/pSNL C57BL/6 mice after the NOX2 inhibitor,

gp91ds-tat (10 mg/kg, i.p.) or vehicle (scrambled) (n=6, ***P<0.001 pSNL scrambled, *vs.* sham scrambled; two-way ANOVA followed by Bonferroni post hoc analyses and one-way ANOVA followed by Bonferroni post hoc analyses); **(e,g)** NOX2 and NOX4 mRNA relative expression in sciatic nerve of C57BL/6 mice after perineural NOX2 and NOX4 AS-ODN or scrambled (all, 10 nmol/10 µl) (n=3 replicates from 2 independent experiments, *P<0.05 AS-ODN *vs.* scrambled, MM-ODN; unpaired two-tailed Student's t-test). **(f,h)** Mechanical allodynia, number of F4/80$^+$ cells, and H_2O_2 content in sham/pSNL C57BL/6 mice after perineural NOX2 and NOX4 AS-ODN or scrambled and MM-ODN (all, 10 nmol/10 µl) (n=6, ***P<0.001 pSNL scrambled, pSNL AS-ODN *vs.* sham scrambled, sham MM-ODN; two-way ANOVA followed by Bonferroni post hoc analyses and one-way ANOVA followed by Bonferroni post hoc analyses). Data are represented as mean±s.e.m.

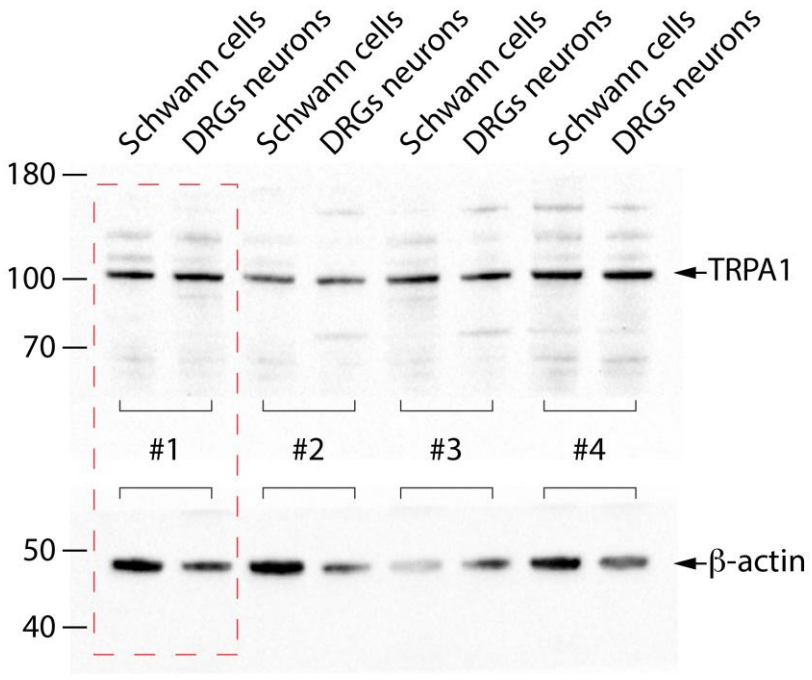

Supplementary Figure 7. The uncropped scan of the blot. The blot area within dashed line box is shown in the figure 3g. Samples have been obtained from 4 different mice.
